# The smell of hunger: Norway rats provision social partners based on odour cues of need

**DOI:** 10.1371/journal.pbio.3000628

**Published:** 2020-03-24

**Authors:** Karin Schneeberger, Gregory Röder, Michael Taborsky

**Affiliations:** 1 Institute for Ecology and Evolution, Behavioural Ecology Division, University of Bern, Bern, Switzerland; 2 Animal Ecology Group, Institute of Biochemistry and Biology, University of Potsdam, Potsdam, Germany; 3 Laboratory of Fundamental and Applied Research in Chemical Ecology (FARCE), Institute of Biology, University of Neuchâtel, Neuchâtel, Switzerland; University of St Andrews, UNITED KINGDOM

## Abstract

When individuals exchange helpful acts reciprocally, increasing the benefit of the receiver can enhance its propensity to return a favour, as pay-offs are typically correlated in iterated interactions. Therefore, reciprocally cooperating animals should consider the relative benefit for the receiver when deciding to help a conspecific. Norway rats (*Rattus norvegicus*) exchange food reciprocally and thereby take into account both the cost of helping and the potential benefit to the receiver. By using a variant of the sequential iterated prisoner’s dilemma paradigm, we show that rats may determine the need of another individual by olfactory cues alone. In an experimental food-exchange task, test subjects were provided with odour cues from hungry or satiated conspecifics located in a different room. Our results show that wild-type Norway rats provide help to a stooge quicker when they receive odour cues from a hungry rather than from a satiated conspecific. Using chemical analysis by gas chromatography-mass spectrometry (GC-MS), we identify seven volatile organic compounds that differ in their abundance between hungry and satiated rats. Combined, this “smell of hunger” can apparently serve as a reliable cue of need in reciprocal cooperation, which supports the hypothesis of honest signalling.

## Abstract 2

Rats provide more food to hungry fellow rats than to satiated ones. This study shows that a rat can assess the need of their partner using odor cues alone, and identifies the volatile organic compounds responsible for this "smell of hunger," a potential example of honest signalling.

## Introduction

Reciprocal cooperation among unrelated individuals is widespread in animals (for a review including 79 vertebrate species, see the work by Taborsky and colleagues [1]). Theoretical models include both the costs to the donor and the benefit for the receiver as important parameters for the evolution of cooperative behaviour [2–4]. The propensity of an animal to cooperate with a conspecific should thus increase when the benefit for the partner is high, while the costs for the helpful individual are low [5]. Animals show need, for instance, for food, by solicitation, which increases the propensity to receive help [6]. Solicitation, for example, by begging behaviour of young vertebrates, frequently involves costly auditory and visual signals [7]. When adult animals cooperate, for example, when individuals are close to a desirable resource but unable to access it, prospective recipients may demonstrate their motivation to get the resource by reaching towards it or by vocalizing (for example, the work by Schweinfurth and Taborsky, the work by Burkart and colleagues, the work by Cronin and colleagues, the work by Yamamoto and colleagues, and the work by Melis and colleagues [6,8–11]). However, vocalisation and gestures may not necessarily reflect the relative need of the recipient honestly but might instead be used to manipulate a potential donor into helping, for example, by attempting to attract attention of the conspecific [11], threaten it [12], or pretend unwarranted need [13], which may reflect dishonesty [14,15] (see the work by Riehl and Frederickson [16] for review). Here, we use wild-type Norway rats (*Rattus norvegicus*) to investigate odour as potentially honest, uncheatable cue for prospective donors to assess the need for food of a conspecific.

Norway rats show both direct and generalised reciprocity in an instrumental reciprocal task, in which one individual can pull food for its partner [12,13,17–19]. Thereby, rats are sensitive to the quality of help they received [18], and they accept higher costs when providing food to a hungry partner in weak condition than to a well-fed one [13]. When rats are able to donate food to a partner in an experimental task, prospective receivers show solicitation behaviours, including reaching toward the desired food, calling in the ultrasonic frequency range, and trying to catch the attention of the donor [6]. These behaviours increase the partner’s propensity to help. It is yet unclear, however, which information is actually transmitted and which sensory modalities are involved in this communication. Furthermore, these signals might be prone to manipulation, that is, to dishonestly feigning urgent need. In order to assess the true need of another individual, a potential donor should only rely on cues that are honest, either because they cannot be easily manipulated or are costly to produce [5,20]. In nocturnal social species such as Norway rats, visual signals are of minor importance and thus should be replaced by other cues [12]. If food is the desired resource, olfaction is a sensory modality that might provide reliable information about the need of an individual. Uncheatable information may be transmitted via inadvertent odour cues resulting from the condition of an animal, for example, its current nutritional state. In rats, carbon disulfide from the breath of conspecifics plays a role in diet choice [21], with rats preferring novel food that has previously been fed on by other rats [22].

Based on this argument, we hypothesised that rats can distinguish between hungry and satiated conspecifics solely by means of odour cues and that they adjust their amount of help toward them accordingly. We used a setup where focal rats could pull food for a stooge in an adjacent compartment while being provided with odour cues from a hungry or satiated rat located in a different room. Thereby, visual and acoustic stimuli from the rat providing odour cues were excluded.

## Results and discussion

### Response to olfactory cues

**Fig 1:**
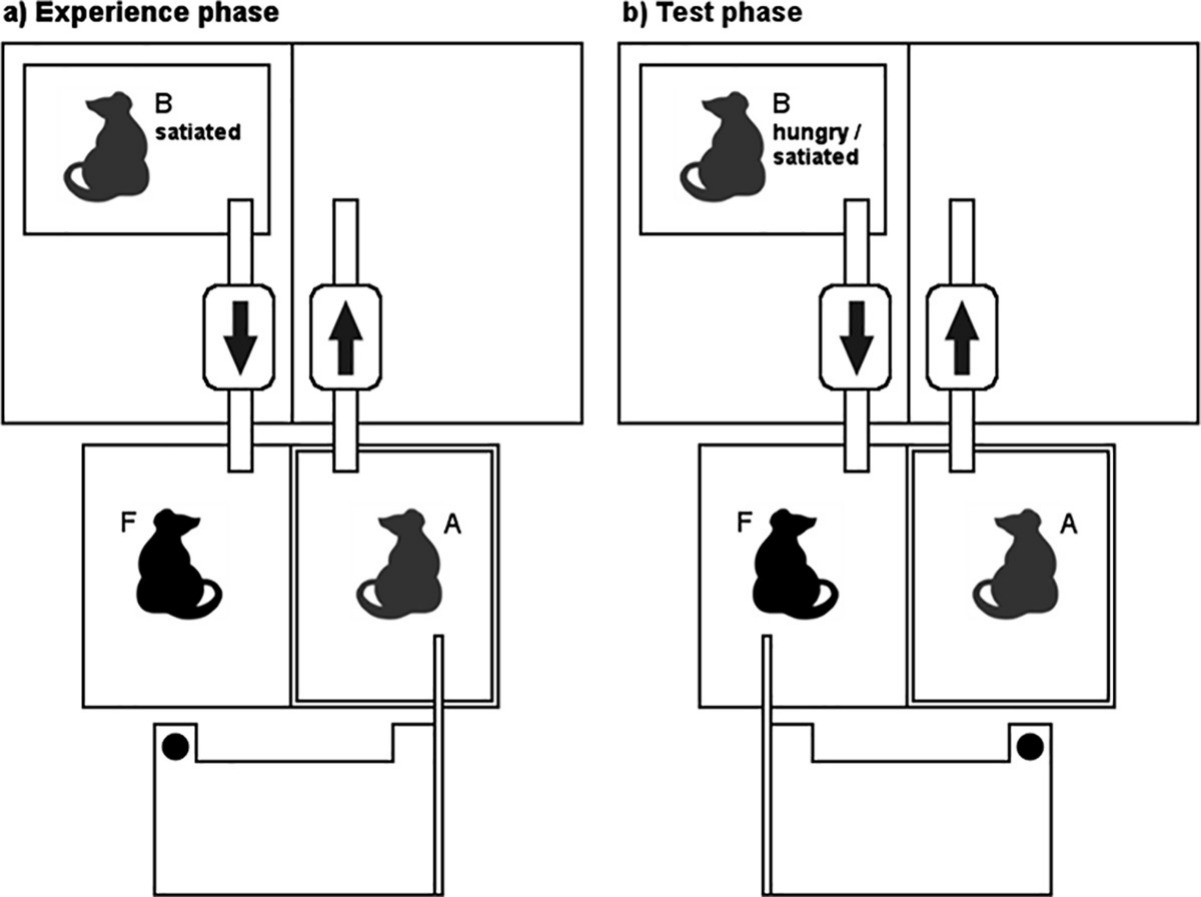
Experimental setup. (a) During the experience phase a cooperative partner (A) placed in a Plexiglas box in the neighbouring compartment of the focal rat (F) produced food for the latter by pulling a stick attached to a tray containing a food reward. The air of the Plexiglas box of cooperator A was removed from the experimental room by a pump (depicted by outgoing arrow). A second individual (B) was placed in a similar box in a different room, and the air from this box was pumped into the compartment of the focal rat (depicted by ingoing arrow). Individual B was satiated during this experience phase. (b) Experimental setup of the test phase. The focal rat now had the opportunity to reciprocate the help it had received during the experience phase by pulling food for the partner in the adjacent Plexiglass box, while receiving odour from a hungry or satiated individual (B) located in a Plexiglas box in another room. The arrows depict air flow as in panel (a).

The experimental setup was adapted from previous experiments on reciprocal food exchange in rats [13,18,19] (Fig 1). A wire mesh divided the test cage (80 cm × 50 cm × 37.5 cm), with one compartment used for the focal rat and the other one for its experimental partner. A movable tray was installed in front of the cage, which could be pulled towards the cage by an attached stick accessible only to one of the two rats. A food reward was placed on the other side of the tray, so that it could be reached only by the experimental partner of the pulling rat, that is, the receiver. Thus, rats could not pull food for themselves but only for their respective partner. Pulling the tray towards the partner is costly for the focal subject, and it has been shown that they take these costs into account when deciding to donate food to a partner [13]. Experimental dyads consisted of unrelated and unfamiliar females. The 20 focal rats used in this experiment had all been made acquainted with reciprocal cooperation and were familiar with the setup (cf., the work by Rutte and Taborsky [17]). Furthermore, we used four rats that were specifically trained to always pull as cooperative partners of the focal test subjects (experimentally assigned ‘cooperators’).

In order to block information transfer between the experimental rats in the adjacent compartments of the test cage, the partner of the focal rat (‘cooperator A’) was placed in a clear Plexiglas box (38 cm × 48 cm × 36 cm) from which a pump sucked out the air and blew it outside of the room. Hence the focal rat was unable to smell the partner in the neighbouring compartment. The air pump created noise in the audible and ultrasonic acoustic ranges of rats, thereby impeding acoustical communication, which was further hampered by the fact that the partner was contained in a sealed Plexiglas box [23]. We furthermore covered the side of the partner rat’s box adjacent to the compartment of the focal individual with opaque foil so that the rats could not see each other.

In the experience phase (Fig 1A), the partner (‘cooperator A’) pulled food for the focal rat during 7 minutes. We noted the latency until the first pulling, as well as the number of pulls. During this experience phase, the focal rat did not receive chemical information from its pulling partner in the neighbouring Plexiglas box but instead was provided with odour from a similar box located in a different room, which contained a second (satiated) rat (‘B’). On the next day, during the test phase (Fig 1B), the focal rat was enabled to reciprocate the help received on the previous day to ‘cooperator A’ while receiving odour from rat ‘B’ in the neighbouring room. Rat ‘B’ had been either food deprived (hungry) or not (satiated) overnight. The experience and test phases were repeated once with opposite hunger states of the individual (B) from which the odour was presented to the focal rat in the test phase. The partners as well as the sequence in which the focal rat received odour from hungry or satiated individuals were randomised. In the test phase, hungry partners were only presented in the morning because of ethical reasons, in order to avoid suffering from prolonged food restriction.

**Fig 2:**
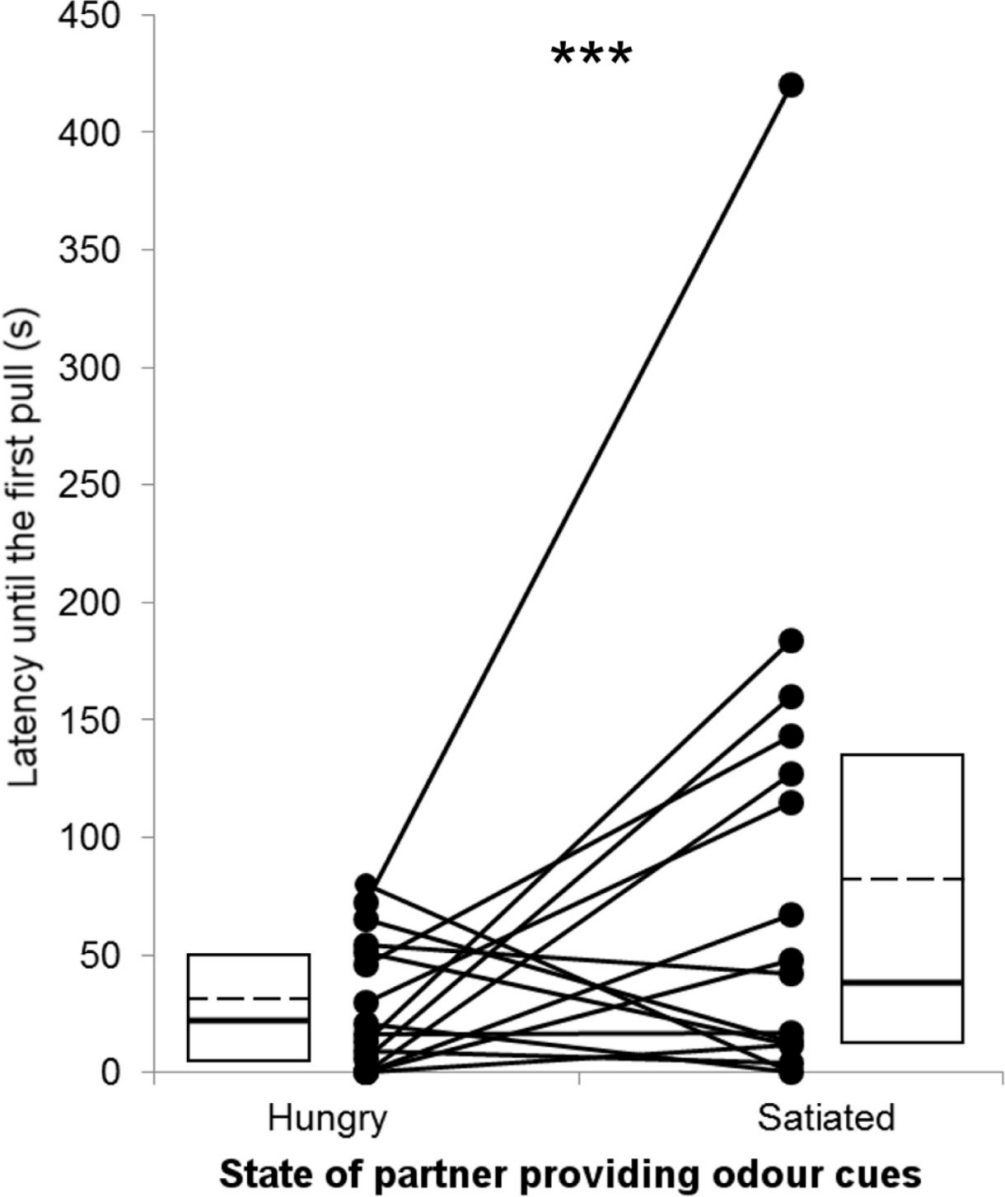
Latency to the start of food provisioning of test rats for a stooge in the neighbouring compartment when receiving odour from a hungry or a satiated conspecific located in a different room (for raw data, see S1 Data). Boxes mark medians (bold line), means (dashed line), and interquartile ranges of the data. The asteriks indicate a significant difference at *p* < 0.001.

Focal rats started to pull earlier for the stooge if they received odour from a hungry rather than from a satiated rat (X^2^ = 413.5; *p* < 0.001; mean latencies for hungry rats: 29 s; for satiated rats: 85 s; *N* = 16; Fig 2), even when excluding the outlier (X^2^ = 191.93; *p* < 0.001; mean latencies for hungry rats: 29 s; for satiated rats: 63 s; *N* = 15; outlier defined as value exceeding mean + (SD × 2)), suggesting that they assess the hunger status of a partner using olfactory cues alone. An enhanced propensity to donate food to hungry as compared with satiated partners has been identified also in previous studies using the same setup, in which the hungry or satiated partners were located in the same cage as the test subject [6,13]. One might argue that olfactory cues emitted by hungry rats could lead to increased general agitation by the focal rat, thereby resulting in higher activity and consequently earlier pulling. However, rats also pull earlier for cooperative than for defective partners [12,13,17,19,24], and the latency to pull correlates negatively with pulling frequency, suggesting that this latency indeed represents the helping motivation of the focal subjects [6,18].

In a previous study, in which focal rats had full access to all cues provided by the partner (visual, olfactory, and acoustic), the mean latency to pull was 196 s for hungry and 183 s for satiated rats [13]. Thus, in the current experiment, in which focal subjects received olfactory cues alone, they started to pull much earlier, and they differed in their latency to pull depending on the hunger state of the receiver. Confining the information received to one modality, olfaction, apparently triggered an earlier response. The lack of pronounced latency differences between pulling for hungry and satiated rats in the previous study, in which the partner was physically present nearby, might indicate that the behaviour of the partner can influence the decision of the focal rat, which has been shown to occur in a previous experiment [6]. We should like to stress that the previous data were obtained with a different batch of rats, and thus the absolute latency and number of pulls may not be directly comparable between experiments because of potential differences between populations (for example, in the general motivation and/or ability to pull).

The mean numbers of pulls by focal rats appeared to be higher when receiving odour from a hungry than from a satiated partner, but this was not significant, and thus there was no evidence that this difference was meaningful (X^2^ = 1.62; *p* = 0.20; mean number of pulls per 7 minutes for hungry rats: 7.56; for satiated rats: 6.37; *N* = 16). In a previous study in which the partner was physically present nearby and no sensory modality was excluded, body mass of the partners in connection with their hunger state played a crucial role in the decision of the focal rat on how much food to provide. When the receiver was hungry, focal rats pulled more often for light than for heavy partners. In contrast, when the receiver was satiated, focal rats pulled more often for heavy than for the light partners. As weight reflects dominance in rats [25], heavier partners may generally more readily receive food donations from focal rats simply because of dominance effects [26]. As the rats in our study were all of similar body weight, dominance can be ruled out as potential explanatory factor for our results.

The latencies until the first pull for the focal rat that the ‘cooperator A’ had shown on the experience day and that of the focal rat pulling for the stooge on the test day were not correlated with each other (rho = 0.027; *p* = 0.85) nor were their numbers of pulls (rho = 0.035; *p* = 0.89). This indicates that the test subjects did not merely copy the behaviour of their partner [23]. During the experience phase, experimental partners pulled earlier and more often for focal rats in the afternoon than in the morning (latency: X^2^ = 67.46; *p* < 0.001; number of pulls: X^2^ = 19.68; *p* < 0.001; *N* = 16), revealing an increase in the motivation of rats to donate food during the course of the day. This contrasts with the behaviour of the focal rats on respective test days; they showed shorter latencies before starting to pull for their partners in the morning (for hungry partners) than in the afternoon (for satiated partners; see above results). Thus, the discrimination between pulling for hungry and satiated partners might be even stronger than observed considering the apparently increasing motivation of rats to pull during the course of the day. Furthermore, we tested for a potential sequence effect by comparing for each individual the number of pulls and the latency to pull between the two test days, which did not reveal a significant effect (paired Wilcoxon signed rank test; number of pulls: V = 99.5; *p* = 0.110; latency: V = 50.5; *p* = 0.379).

A previous study of Norway rats showed that they reciprocate help according to the quality of help they previously received [18], indicating that the specific outcome of a helpful act for the receiver can subsequently benefit the donor. In our study, rats provided food faster when the partner was hungry than when it was satiated. ‘Gratitude’ is often argued to play a key role in the evolution of social behaviour in humans, with individuals being more willing to help somebody else if they are grateful for the help they have previously obtained [27]. If individuals reward cooperative conspecifics according to their perceived benefit of previously received help, assessment of the need of the partner might critically affect the probability to get help back in a future interaction.

In our experiment, the rat from which the odour derived was separated from the experimental room and placed in a box alone. Hence, the information transferred by the odour was not socially induced. Therefore, rats seem to use the partner’s inadvertent smell of hunger as a reliable indicator for its current need, adjusting their helping propensity accordingly. This supports the ‘index hypothesis’ of honest signalling, which assumes that honesty is enforced because of physical, developmental, or physiological constraints that cannot be cheated [5,20].

### Odour components

Our experimental results above suggested that distinct volatile organic compounds (VOCs) might differ between hungry and satiated rats. We thus analysed olfactory samples taken from hungry and satiated rats for within-individual comparison of emitted volatile olfactory compounds.

**Fig 3:**
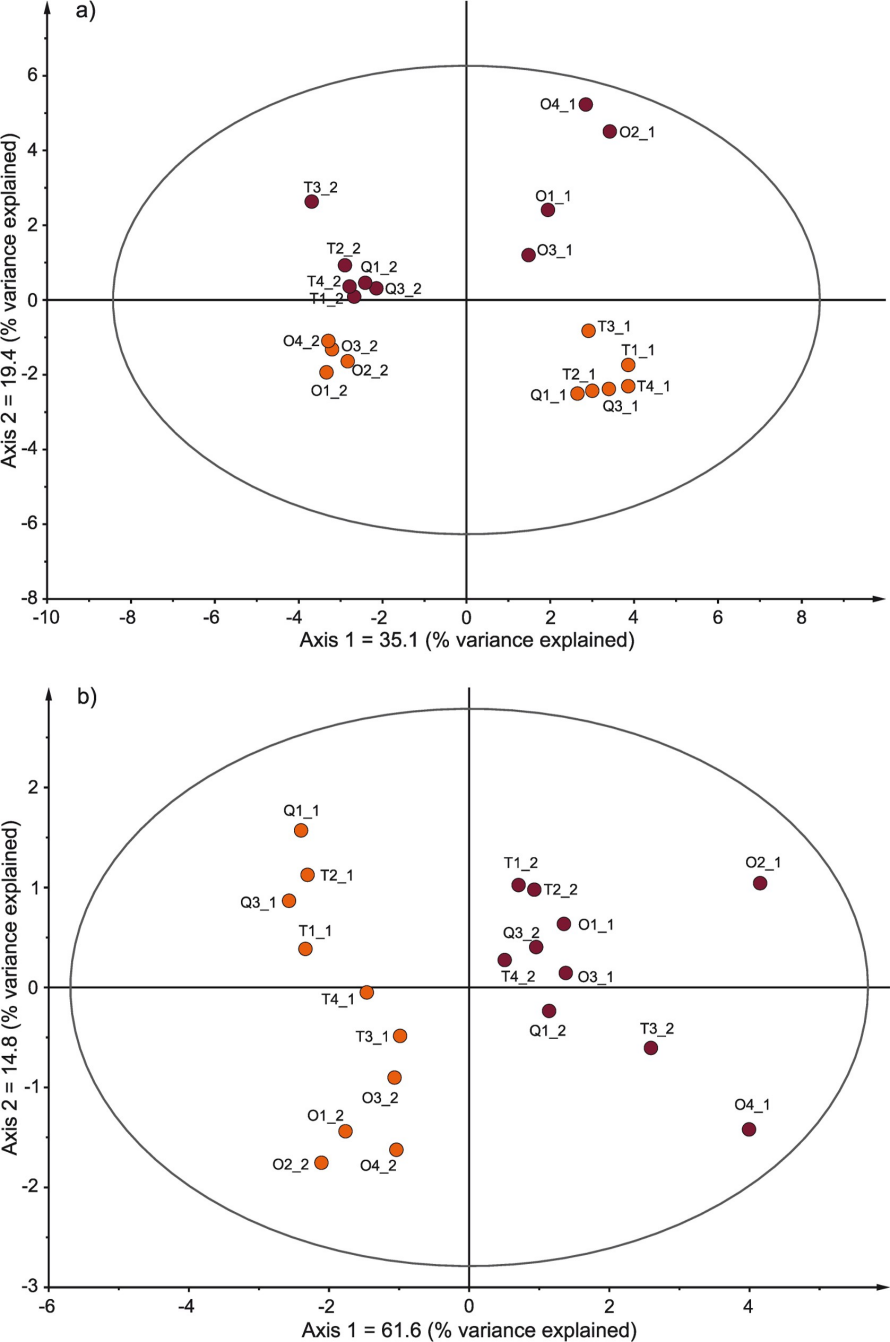
**Principal component analysis of 27 biologically relevant olfactory compounds (a) and the 7 significantly different compounds (b) in the odour of hungry (orange/light) and satiated (purple/dark) rats (for raw data, see S2 Data).** Ellipses mark Hotelling's T2 (95%). Dots are marked with individual IDs, with individuals with the same letter in the name being housed in the same group, and the numbers behind underscores depicting the sample sequence (_1 or _2).

For this experiment, a hungry or satiated rat was individually placed in a Plexiglas box from which the air was sucked out with a vacuum pump (Intex®110 quick fill, 400 L/min). This air was trapped in an adsorbent filter, which was later analysed using a gas chromatography system coupled with a mass spectrometer (see Methods). A principal component analysis (PCA) of 27 biologically relevant volatile olfactory compounds identified in the air collected from the experimental rats’ box revealed that the olfactory profiles cluster according to the hungers status of the individuals (Fig 3A). Significance of the model has been assessed using both goodness of fit (R^2^ = 0.665) and goodness of prediction (Q^2^ = 0.267) parameters. Seven compounds were significantly different between hungry and satiated rats (*N* = 10; Table 1; Q^2^ = 0.369; Fig 3B; S1 Fig): propanoic acid, butanoic acid, butyl acetate, 3-methylbutanoic acid, pentanoic acid, 2-heptanone, and dimethyl sulfone. Notably, butyl acetate and butanoic acid were present only in hungry rats, whereas pentanoic acid was detected only in satiated individuals.

**Table 1:** Twenty-seven biologically relevant volatile organic compounds in rat odour. Given are retention times (RT), mean relative abundance (a compound’s peak area in chromatographic profile) for hungry and satiated rats, the mean difference in abundance, and test statistics for each compound using 5,000 permutations and Bonferroni correction for multiple testing. Substances that differed significantly in their abundance between hungry and satiated rats are marked in grey. Formally identified compounds are marked in bold.

Compound	RT	Mean abundance			F	*p*
		Hungry rats	Satiated rats	Difference		
**Acetic acid**	1.720	6,335,471	5,882,134.6	453,336.4	0.151	0.712
**Propanoic acid**	2.250	1,319,250.1	2,182,683.6	−863,433.5	12.57	<0.001
**Butanoic acid**	3.150	1,580,599.8	2,460,690.3	−880,090.5	11.60	0.002
Hexanal	3.231	1,167,781	1,068,713.778	99,067.22222	2.16	0.176
**Butyl acetate**	3.451	380,427.8889	0	380,427.8889	46.77	<0.001
**3-Methylbutanoic acid**	3.904	187,862	0	187,862	11.67	0.009
**Pentanoic acid**	4.671	0	382,177	−382,177	16.22	<0.001
**2-Heptanone**	4.830	585,068.25	1,322,360.1	−737,291.85	16.21	<0.001
**Heptanal**	5.047	2,643,706	3,434,022.9	−790,316.9	0.752	0.401
**Dimethyl sulfone**	5.493	693,179.8	1,674,063.1	−980,883.3	20.44	<0.001
**Hexanoic acid**	6.895	1,344,355.4	1,581,008.6	−236,653.2	0.619	0.437
**5-Hepten-2-one, 6-methyl-**	7.070	5,212,116.75	4,861,344.667	350,772.0833	0.465	0.503
Octanal	7.474	673,047	653,348	19,699	0.107	0.758
**3-Carene**	7.656	844,680.75	424,704.4444	419,976.3056	4.16	0.057
2-Hexanone, 3-methyl-	8.199	1,260,886.714	982,749.625	278,137.0893	0.117	0.740
Benzoic acid, 4-methyl-, 2-hydroxy-2-phenylpropyl ester	9.678	558,842.625	459,576.6667	99,265.95833	0.093	0.764
2-Penten-1-ol	9.876	1,277,454.5	1,003,322	274,132.5	3.65	0.069
Nonanal	10.196	2,576,800.7	3,278,869.1	−702,068.4	0.740	0.400
**Dodecane**	12.824	759,666.7	620,910.75	138,755.95	3.32	0.085
Decanal	12.986	1,623,969.6	1,843,755.2	−219,785.6	0.207	0.647
Butanoic acid, 3 hydroxy-3-methyl	14.000	543,081.625	464,247.5556	78,834.06944	0.025	0.875
**Tetradecane**	18.116	1,399,337.8	1,205,279.5	194,058.3	1.50	0.225
**Caryophyllene**	18.654	868,109.8333	788,356.25	79,753.58333	1.11	0.270
5,9 Undecadien-2-one, 6,10-dimethyl-(E)	19.452	2,431,564.5	2,483,257.167	-51,692.66667	0.751	0.380
**Nonadecane**	22.923	1,938,723.1	1,841,207.8	97,515.3	0.120	0.734
**n-Hexadecanoic acid**	32.222	4,400,507.625	4,111,716.111	288,791.5139	0.020	0.884
**Octadecanoic acid**	37.860	1,888,030.429	1,860,893.222	27,137.20635	0.419	0.525

**Abbreviations:** RT, retention times

Propanoic and butanoic acid quantities were higher in satiated than in hungry rats. These organic compounds are biosynthesised in the large intestines by bacterial fermentation of dietary fibre [28]. Similarly, the dimethyl sulfone amount was higher in satiated than in hungry rats, which is a metabolite of dimethyl sulfoxide that is found in many foods, including grains and raw vegetables, both of which are regularly fed to our rats [29]. As hungry rats did not have access to food for 16 h, this fasting condition may have been reflected by their low abundance of dimethyl sulfone. Thus, low emanation of these 3 substances in hungry rats may be directly linked to reduced access, intake, and digestion of food and therefore serve as reliable indicators of hunger. 2-Heptanone, which occurs in certain foods as well, was also more abundant in the odour of satiated than hungry rats. This organic compound is contained in high concentration also in the urine of physically stressed rats, potentially serving as alarm cue for conspecifics [30]. However, the 2-heptanone amount was lower in hungry than in satiated rats; hence, in our satiated rats, the quantity of this substance may have indicated a relatively high metabolic activity rather than stress, which should have reached higher levels when the rats were hungry.

Of highest significance may have been the 3 substances that were present only either in samples from hungry or satiated rats. Pentanoic (or valeric) acid that was detected only in satiated individuals is a carboxylic acid produced by gut bacteria in the colon of rats [31]. Thus, this substance may be directly connected to metabolic activity. In contrast, butyl acetate and 3-methylbutanoic acid were present only in the odour of hungry rats. Butyl acetate can be found in fruits such as apples but also in building material such as wood. As the rats did not receive fruits during our experiment, it is possible that the hungry rats might have gnawed on the wooden enrichment in their home cages during the food restriction. Interestingly, 3-methylbutanoic acid is part of the female pheromone blend of rats [32], the specific functions of which in rat communication are hitherto unknown, however. Future research might clarify what information is precisely transferred by the release of 3-methylbutanoic acid that could be responsible for the increased helpfulness of conspecifics.

### Implications

Our results show that Norway rats adjust their propensity to help to the current need of a partner, apparently using olfactory cues either resulting directly from recently ingested food sources, from metabolic processes involved in digestion, or from a putative pheromone indicating hunger. It is likely that these cues cannot be cheated, thereby providing reliable information about one’s need for potential donors [5,20]. This may illustrate a simple mechanism by which individuals can make adaptive social decisions if their interests are correlated through iterated interactions [1].

Norway rats are not the only species where unrelated individuals share food with each other reciprocally. In the common vampire bat (*Desmodus rotundus*), conspecifics regurgitate blood for individuals that hunted unsuccessfully, thus saving them from starvation [33]. A recent study suggesting that in these exchanges the bats apply decision rules of direct or generalized reciprocity [34] showed that food sharing was preceded by allogrooming and sniffing, which might indicate that odour plays an important role in decision making also in this species [35]. Olfactory cues may potentially inform the donor about whether the receiver has been hunting successfully or not. Furthermore, odour might be used also as a signal when needing help in a cooperative task, as has been suggested for elephants [36]. In any case, odour could provide important information to assess the need of a partner for help in many species showing food provisioning. In primates, including humans, in which food sharing amongst unrelated individuals is common (reviewed in the works by Jaeggi and Gurven [37,38]), odour is unlikely to serve as a cue for need, because visual and acoustic cues are ecologically more relevant for this order. The relative amount of food shared is usually correlated with the number of consumers within the recipient family. In humans, it is higher for young and old than for middle-aged individuals, with food items having a higher relative caloric value to these favoured receivers [39]. This might have long-term benefits for the donor as well, because in a species showing reciprocal cooperation, generous individuals can rely on receiving help back when being in need themselves [40], especially if the conflict of interest is low [6]. In humans, the control of reciprocal cooperation may involve social norms and their implementation, which can be based on uncheatable cues of need and may include punishment of cheaters [39].

## Methods

### Ethic statement

Housing of the animals and the experimental procedure were authorised by the Swiss Federal Veterinary Office (licence no. BE25/14) in accordance with the animal welfare regulations of Switzerland (Tierschutzverordnung Schweiz 04/2008).

### Experimental subjects and housing

Female wild-type Norway rats (*Rattus norvegicus*; source: Animal Physiology Department, University of Groningen, the Netherlands) were housed in groups of 5 sisters each in environmentally enriched cages (80 cm × 50 cm × 37.5 cm; in accordance with animal welfare legislation of Switzerland [41]), with commercial rat pellets and water provided ad libitum. Housing room conditions were held constant at 20°C ± 1°C and 45% to 55% humidity under a 12:12 h light-dark cycle with lights on at 8 p.m. We performed experiments during the dark phase under red light, because rats are primarily nocturnal and lack receptors for red light. All rats were individually marked and used to frequent handling to ensure that they were not stressed during the experiment [6,12,13,17–19]. During the experiment, the latency until the first pulling was used as the critical response variable, because this is a continuous variable containing more information than the integer pulling frequency, and it has been shown to be a reliable indicator for the rats’ motivation to help [6,18].

### Chemical analyses

Before taking an odour sample, the Plexiglas box was cleaned with ethanol and the hungry or satiated rat placed in the box. Then, an adsorbent tube filled with 70 to 75 mg Tenax-TA porous polymer (Gerstel GmBH) was fixed about 15 cm above the animal. A 5-min collection period was used to trap the emitted volatiles by connecting the adsorbent tube to a diaphragm vacuum pump with a flexible hose (Vacuubrand GmBH, 3,000 mL/min suction flow). Afterwards, the filters were stored in individual 8-mL amber glass vials hermetically closed and kept at 10°C before being analysed on the same day.

A gas chromatography system (GC, Agilent 7890a) coupled with a mass spectrometer detector (MSD, Agilent 5975c) was used for identification and relative quantification of the volatile compounds released by hungry or satiated rats. Tenax filters were desorbed in a thermal desorption unit initially at 40°C (held for 0.5 min), then ramped 60°C/min until 250°C (hold time 3 min). During this step, all the compounds were cryofocused with liquid nitrogen in a cooled injection system (−80°C, held for 8 min) before being released (12°C/min ramp, 280°C final temperature). A PTV inlet was used in solvent vent mode for injection (pressure 14 psi = 96.5 kPa, total flow 51.52 mL/min). Compounds were separated on an Agilent HP5-MS column (30 m length × 0.25 mm i.d., and 0.25 μm film thickness) undergoing an initial 50°C (1 min), then a first ramp of 5°C/min until 160°C, a second of 3°C/min to 200°C, and finally raised 100°C/min until 250°C (hold time 3 min). Helium was used as carrier gas, at a 1.65 mL/min flow rate (constant flow mode). A 2 min post run at 260°C was performed between each sample. The MSD transfer line was set at 280°C. In the MS detector, an electron impact mode (70 eV) and a scanning over de mass range of 33 to 300 were used. Corresponding blank (empty glass tube) and controls (odours collected in empty cages, one per sampling day) were carried out in order to determine which of the compounds were not of rat origin. No exact quantification (internal standard or calibration curve) was undertaken in this study.

All chromatographic profiles were manually processed in order to exclude likely contaminants and irrelevant volatiles. Preliminary identifications of the VOCs were based on NIST11 mass spectral library as well as PBM library search (U.S. Department of Commerce and Agilent Technologies, Inc.). Using SciFinder^®^, 27 obvious and biologically relevant VOCs were selected for further statistical analysis. Auto-integrations were carried out to obtain relative abundances in all chromatograms. In order to confirm the identities of each of the 18 main compounds, we analyzed pure standards (Merck—Sigma-Aldrich, KGaA) for each of them with the exact same analytical procedure. In all cases, retention times and mass spectra comparisons confirmed their initial identifications (see Table 1), including all of the 7 volatile components that significantly differ between hungry and satiated rats.

### Statistical analyses

We analysed the pulling behaviour of the focal rat with generalised linear mixed models (GLMM) using R statistical software (version 3.1.3; http://www.r-project.org) and the package ‘lme4’. We tested for the effect of the hunger status of the partner from which the focal rat perceived the odour on the latency to the first pull and the number of pulls using GLMMs with Poisson distributions and comparing them to null-models. The identities of (i) the focal rat, (ii) the cooperative partner in the adjacent compartment, and (iii) the rat in the neighbouring room from which the odour derived were included as random factors. In order to exclude a sequence effect as potential confounding factor, we tested whether the motivation to perform the pulling tasks differs between morning and afternoon, using data obtained from cooperators during the experience phase. We performed GLMMs using time of the day as explanatory variable as well as cooperator identity as random factor to explain the latency until the first pull and the number of pulls during 7 min. Additionally, we tested if the latency and number of pulls of the focal rats correlated with the previous performance of the cooperator during the experience phase using Spearman rank correlation tests.

Based on their relative abundances, a PCA was carried out on the 27 biological relevant VOCs. Using Simca 13.0 software (Umetrics, Sartorius Stedim Biotech, Sweden), the data were converted into visual information for interpretation. Furthermore, relative abundances of the identified substances were compared between hungry and satiated rats using a linear model. Most of the data failed the assumptions of normality and equality of variances, thus the F-values were obtained from the original model whereas *p*-values were obtained by 5,000 permutations (see S1 Script for the R script). A Bonferroni *p*-value adjustment method was applied to account for multiple testing.

## Supporting information

S1 Fig Sums of VOCs collected from either hungry (white) and satiated (black) rats (for raw data see S2 Data). Relative abundance is measured in number of ions. Significant differences are marked with an asterisk. VOC, volatile organic compound
(PDF)

S1 Data Raw data of rat performance. (XLSX)

S2 Data Raw data of GCMS output. GCMS, gas chromatography–mass spectrometry
(XLSX)

S1 Script R Script to obtain *p*-values for the differences in relative substance abundance between hungry and satiated rats. (TXT)

## Abbreviations

GC-MS: gas chromatography-mass spectrometry
GLMM: generalised linear mixed models
PCA: principal component analysis
VOC: volatile organic compound
