# Supplementary material for: The smell of hunger: Norway rats provision social partners based on odour cues of need
Source: PLoS Biol. 2020 Mar 24;18(3):e3000628. doi: 10.1371/journal.pbio.3000628 (PMC7092957; doi:10.1371/journal.pbio.3000628)
Supplement: S1 Fig — Relative abundance is measured in number of ions. Significant differences are marked with an asterisk. VOC, volatile organic compound (PDF) [file pbio.3000628.s001.pdf]

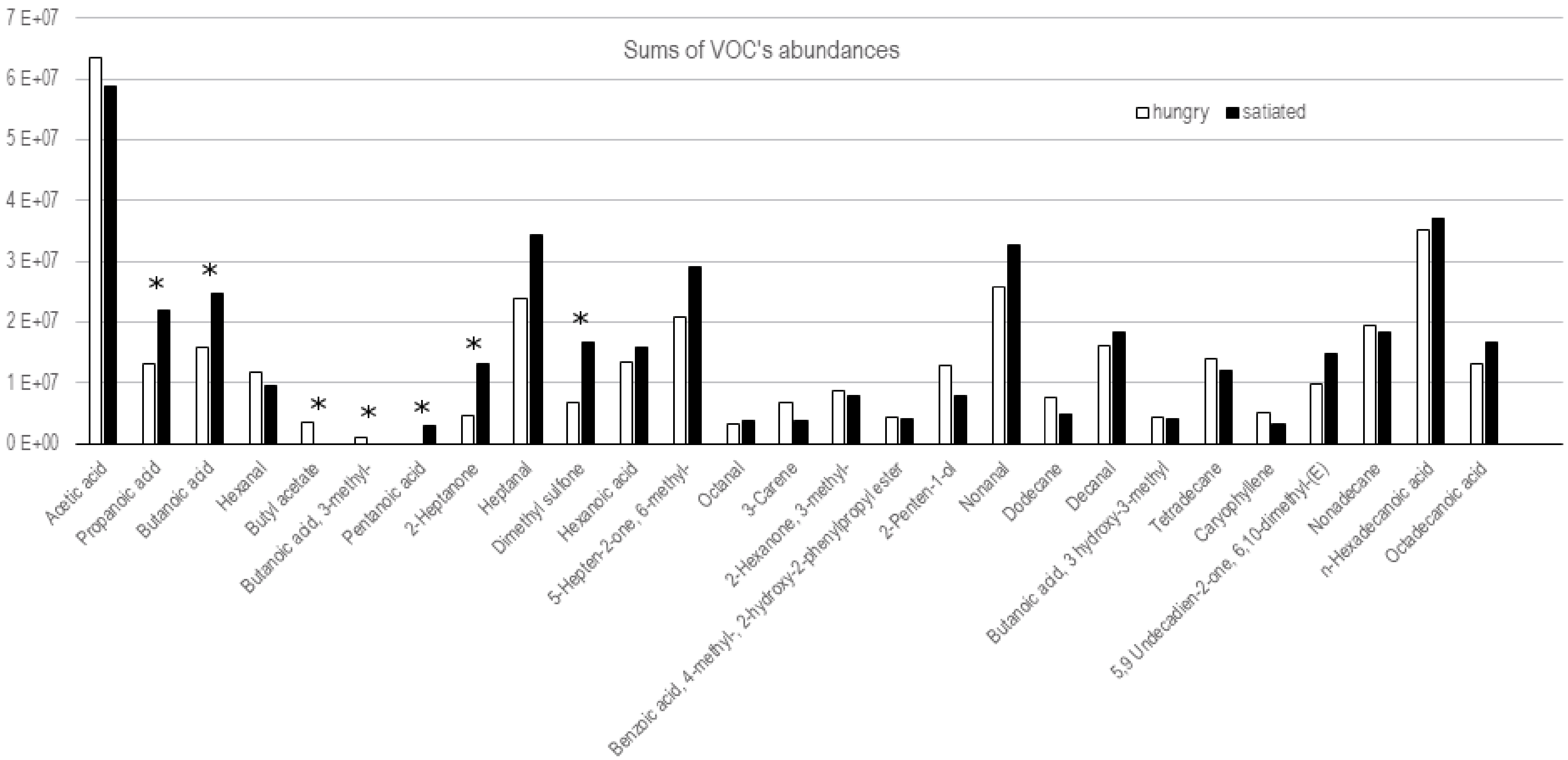

Supporting Information Figure 1: Sums of volatile organic compounds (VOCs) collected from either hungry (white) and satiated (black) rats (for raw data see S2_Data). Relative abundance is measured in number of ions. Significant differences are marked with an asterisk.
